# Getting Interdisciplinary Teams into the Field: Institutional Review Board Preapproval and Multi‐Institution Authorization Agreements for Rapid Response Disaster Research

**DOI:** 10.1111/risa.13740

**Published:** 2021-05-07

**Authors:** Lori Peek, Jennifer Tobin, John W. van de Lindt, Anne Andrews

**Affiliations:** ^1^ Department of Sociology and Natural Hazards Center University of Colorado Boulder Boulder CO USA; ^2^ Natural Hazards Center University of Colorado Boulder Boulder CO USA; ^3^ Department of Civil and Environmental Engineering Colorado State University Fort Collins CO USA; ^4^ Research Protections Office National Institute of Standards and Technology Gaithersburg MD USA

**Keywords:** disasters, Institutional Review Board (IRB), interdisciplinary research, longitudinal field study, rapid response disaster research

## Abstract

This article describes an interdisciplinary community resilience research project and presents a case study that supports bringing researchers together before a disaster to develop plans, procedures, and preapproved Institutional Review Board (IRB) protocols. In addition, this article explains how researchers from various academic institutions and their federal agency partners can effectively collaborate by creating an IRB Authorization Agreement (IAA). Such preparations can support interdisciplinary rapid response disaster fieldwork that is timely, ethically informed, and scientifically rigorous. This fieldwork preplanning process can also advance interdisciplinary team formation and data collection efforts over the long term.

## 1 INTRODUCTION

As disasters have increased in frequency and intensity, academic researchers and funders have called for more, and more rigorous, interdisciplinary research (Kendra & Nigg, 2014). Interdisciplinary research—which involves synergy, synthesis, and integration across disciplines and throughout the research process (Klein, 1990)—is crucial to address knowledge gaps, advance the field of hazards and disaster research, and reduce risk to communities (National Research Council, 2006). It remains challenging, however, to organize, train, and coordinate researchers from different disciplines and various institutions in anticipation of a disaster while still ensuring the ethical conduct of postdisaster fieldwork (Peek, Tobin, Adams, Wu, & Mathews, 2020).

A primary goal of rapid response research is to collect perishable data, or “data that will change or be lost over time” (Oulahen, Vogel, & Gouett‐Hanna, 2020, p. 1). While the emphasis is typically on getting into the field quickly, the actual timeframe for collecting perishable data differs across disciplines. For example, engineers might need to deploy within hours or days of an event to conduct rapid damage assessments of buildings, even though they must strive to not interfere with search and rescue or recovery operations. It is necessary for engineers to collect such data before the damaged structures are altered by natural forces or removed as part of debris clearance activities (Wartman et al., 2020). Social scientists tend to have a longer time span for launching their rapid response studies—often weeks or even months after an event. Social science studies can sometimes be delayed because they are usually contingent on receiving institutional approval for human subjects research (Kendra & Gregory, 2019) or because researchers might wait to collect data until they believe disaster survivors are in a better position to respond to interview or survey questions. When working in interdisciplinary teams, it is imperative that the research goals, timelines for collecting perishable data, and ethical standards be established before a disaster in order to ensure that survivors are protected and the integrity of local scientific efforts are preserved (Gaillard & Peek, 2019; Louis‐Charles, Howard, Remy, Nibbs, & Turner, 2020).

This article presents a case study that demonstrates how researchers from different disciplines can come together before a disaster to develop plans, procedures, and preapproved Institutional Review Board (IRB) protocols. The case study highlights field study efforts that followed flooding in Lumberton, North Carolina (van de Lindt et al., 2020; van de Lindt, Peacock, & Mitrani‐Reiser, 2018) and involved researchers from multiple academic institutions and the federal government. It illustrates the importance of having one institution serve as the IRB of record and demonstrates how an IRB Authorization Agreement (IAA) can foster more efficient research collaboration through streamlining the human subjects research review process. The goal of preparing in this way is to ensure that postdisaster fieldwork is timely, ethical, and scientifically rigorous.

## 2 BACKGROUND AND CASE STUDY FOCUS

In 2015, the National Institute of Standards and Technology (NIST) awarded a five‐year grant to establish the Center for Risk‐Based Community Resilience Planning: A NIST‐Funded Center of Excellence (NIST‐CoE). This grant was renewed for an additional five years in 2020. Headquartered at Colorado State University, the NIST‐CoE involves more than 120 researchers and students across 15 universities and spans multiple disciplines within engineering, urban planning, and the social, behavioral, and economic sciences. An additional 12 NIST researchers from the Community Resilience Group and Office of Applied Economics collaborate with members of the CoE on various research endeavors. The project entails extensive analytical modeling activities, testbed applications, and postdisaster field studies (Ellingwood et al., 2016; Guidotti, Gardoni, & Rosenheim, 2019; van de Lindt et al., 2020) that contribute to the Interdependent Networked Community Resilience Modeling Environment (IN‐CORE). IN‐CORE has the capacity to help end users evaluate risks and make risk‐informed hazard mitigation and recovery investment decisions at the community scale. From the outset of the project, experts have worked across disciplinary boundaries to systematically document how physical, economic, and social infrastructure systems interact in complex environments affected by natural hazards (McAllister, 2013).

## 3 INSTITUTIONAL REQUIREMENTS FOR RESEARCH

The postdisaster field studies that are part of the NIST‐CoE project involve the collection of original data to validate IN‐CORE results and strengthen overall community resilience modeling. To complete these field efforts, the full team needed to understand and adhere to various institutional requirements associated with human subjects research before collecting data. This was especially important given the interdisciplinary nature of the project, since many engineers on the team had never engaged in human subjects research; just as many of the social and behavioral scientists had little experience with damage assessment surveys and translating field study findings into a computational environment. To address these disciplinary divides and serve as an exemplar for future interdisciplinary studies, two of the sociologists on the project team prepared a primer on institutional requirements for human subjects research, which we briefly describe below.

### 3.1 The Institutional Review Board

The IRB process is undoubtedly familiar to academics who conduct human subjects research on a regular basis. As research pursuits become increasingly interdisciplinary, however, it is necessary to incorporate IRB‐specific training into team development processes, especially those that include researchers with limited or no experience working on human subjects studies.

Since the 1970s, U.S. universities have mandated that proposals for academic research involving human subjects are reviewed and approved by an IRB before researchers can collect data. The purpose of IRB review and oversight is to protect the rights and welfare of research participants. It also ensures that research is carried out in compliance with the Belmont Report's foundational ethical principles of beneficence, autonomy, and justice (National Institutes of Health, 1979). The IRB process requires that the principal investigator at a university submit a research plan that requests the level of IRB review and describes research activities, such as how participants will be recruited and how data will be collected (see Table I).

**Table I:** Typical IRB Protocol Sections

Protocol Topic	Brief Description of Required Information
Investigator information	Identify the principal investigator and provide information for all additional investigators associated with the project.
Level of IRB review	Identify appropriate level of IRB review: 1) *Exempt review—*poses no more than “minimal risk” and fits with one of the exempt review categories defined by federal regulation; (2) *Expedited review—*poses no more than “minimal risk” and fits with one of the federally‐designated expedited review categories; (3) *Full board review—*presents more than minimal risks to subjects and will receive review at a fully convened IRB committee meeting; (4) *Not human subjects research—*IRB determines a project does not meet the regulatory definitions for human subjects research; a determination letter will be generated which states IRB approval is not required.
Study objectives and empirical or theoretical background	Provide an overview of the study and a brief literature review that offers a rationale for the research. Describe the empirical or theoretical drivers of the research.
Research study design	Describe the methodological approach for participant sampling, data collection, and data analysis.
Human subjects population(s) of focus	Identify the study population and explain the inclusion or exclusion criteria.
Vulnerable populations (e.g., children, pregnant women, incarcerated populations)	Identify vulnerable populations included in the study and discuss safeguards to protect their rights, privacy, and welfare. Discuss how undue influence and/or coercion will be avoided.
Recruitment methods	Explain how participants will be identified and recruited for the study. Include attachments of any recruitment scripts or letters of support from partner organizations that have agreed to support recruitment efforts.
Informed consent	Describe where, when, and how consent will be obtained and discuss what steps will be taken to avoid coercion.
Procedures	Describe how the data will be collected. Include attachments of any research tools being used such as survey instruments, interview guides, or observation protocols.
Risks to participants	Describe all potential risks to participants and detail how risks will be explained to participants and managed.
Potential benefits	Describe any potential benefits to participants or to the broader community or society. If no benefits are envisioned, indicate this as well.
Data management	Describe how the data will be stored and protected, including whether the original data will be destroyed after a period of time.
Protection of participant privacy	Discuss the plans for protection of the privacy of participants. Detail all plans for the deidentification and/or publication of original participant data.

After submission, an institution's designated IRB committee reviews the protocol materials and either approves or disapproves the study based on criteria that considers the balance of risks and benefits associated. For proposals requiring further clarification, researchers have the chance to respond to concerns via a revision and resubmission process.

The IRB process is designed to protect human subjects from harm. However, if multiple researchers from different institutions are involved in large research projects, the IRB protocol and approval process can become complicated and time consuming (Kendra & Gregory, 2019). One study found that research protocols submitted to a single U.S. university for full review resulted in a median time of 39 days from submission to final decision (AAHRPP, 2015). When submitting IRB protocols across multiple institutions, this can result in even longer time delays and conflicting requirements related to institutional and legal mandates. This, in turn, can lead to lengthy and potentially confusing consent forms that place undue burdens on research participants (Browne & Peek, 2014). These challenges are particularly relevant in postdisaster settings, when familiar research ethics issues are amplified as researchers attempt to enter the field rapidly to collect perishable data, potentially from vulnerable populations (Ferreira, Buttell, & Cannon, 2018; Tansey et al., 2017).

### 3.2 Revisions to Human Subjects Research Requirements

The 2018 Code of Federal Regulations (45 CFR 46) was recently updated to simplify the IRB process in response to some of the aforementioned issues. Effective July 19, 2018, the U.S. Department of Health and Human Services, and many other federal departments and agencies, issued revisions to the Common Rule for human subjects research. Changes were intended to “better protect human subjects involved in research, while facilitating valuable research and reducing burden, delay, and ambiguity for investigators” in an effort to “modernize, simplify, and enhance the current system of oversight” (U.S. Department of Health and Human Services, 2018, p. 7149). Institutions were required to begin implementing these updates on January 21, 2019.

Five key revisions to the Common Rule include: (1) updates to continuing review requirements; (2) new categories of research that do not require IRB review; (3) an expansion to the criteria for exempt status; (4) additional consent elements; and (5) a requirement that U.S. institutions engaged in particular forms of cooperative research rely on a single IRB (U.S. Department of Health and Human Services, 2018). These changes reduce oversight requirements for some categories of less risky human subjects research.

### 3.3 Institutional Review Board Authorization Agreements

Per the revised common rule, exempt human subjects research that requires limited IRB review and research projects designated nonexempt are mandated to seek reliance agreements, such as IAAs, when multiple institutions are involved. This process allows one institution to act as the lead reviewing institution for an entire cooperative research project (Winkler, Witte, & Bierer, 2015). Once the lead institution is identified, the other institution(s) involved enter into an agreement to cede IRB review responsibilities to the lead IRB. According to the Office of Human Research Protections, U.S. Department of Health and Human Services, 45 Code of Federal Regulations 46.114:

Cooperative research projects are those projects covered by this policy that involve more than one institution. In the conduct of cooperative research projects, each institution is responsible for safeguarding the rights and welfare of human subjects and for complying with this policy. With the approval of the department or agency head, an institution participating in a cooperative project may enter into a joint review arrangement, rely upon the review of another qualified IRB, or make similar arrangements for avoiding duplication of effort (U.S. Department of Health and Human Services, 2018).

The IAA substantially streamlines review requirements for multiinstitutional efforts and ensures research participants are not burdened with reading and signing multiple separate consent forms before participating in a project. It is important to note, though, that the establishment of an IAA in no way diminishes the ethical responsibility of researchers outside of the lead institution. All researchers, regardless of their institutional home, are still expected to complete required training and adhere to the highest ethical standards.

For the case study described in this article, the Colorado State University IRB agreed to act as the reviewing IRB or IRB of Record for protocol reviews related to collaborative projects under the award (CSU IRB, 2019). NIST and all but one of the partner academic institutions ceded IRB oversight to Colorado State University through signed IAA agreements. In the case of the academic institution that chose not to cede oversight, a second IRB protocol and consent form was required.

### 3.4 Collaborative Institutional Training Initiative

Before academic researchers in the United States can submit IRB protocols or be named as part of a larger multiinstitution IAA effort, they must first complete the required ethics training from a university‐sanctioned institution. A common outlet for such training is the Collaborative Institutional Training Initiative (CITI) program, which offers human subjects research courses for biomedical and social–behavioral–educational research (https://about.citiprogram.org). Before joining a NIST‐CoE‐funded field study, all members of the team who expected to collect data from human subjects, view or analyze the identifiable data collected, or supervise a student in analyzing identifiable data were required to complete the CITI training and submit their course certificates to their home academic institution and the IRB research coordinators. After all the CITI certificates—which are valid for three years—were collected and organized, they were submitted to the Colorado State University IRB as the lead institution.

## 4 PREPARING FOR INTERDISCIPLINARY FIELD WORK

There are several important *institutional processes and requirements* that members of interdisciplinary hazards and disaster research teams must adhere to before conducting rapid response fieldwork. There are also numerous *team‐based processes* that we identified as critical to the formation and successful deployment of our interdisciplinary field team.

### 4.1 Establishing Interdisciplinary Team and Task Leadership

In large and complex projects, it is important to have clear leadership structures to help streamline communications, decision making, and task completion, while still leaving space for the active and creative participation of all team members. To advance interdisciplinary research goals, it is also vital to have representation across disciplines. For these reasons, the initial field study efforts were led by two sociologists, two engineers, and two urban planners. The broader field study task involved researchers from NIST and 11 of the 15 academic institutions involved in the Center of Excellence.

The five primary responsibilities of the field study leadership team are to (1) serve as primary points of contact for communication with the Colorado State University IRB, NIST, and the partner academic institutions; (2) educate researchers about the importance of IRB oversight and ethical approaches to research; (3) encourage and track ethics training completion and ensure all CITI certificates are up to date and in compliance with IRB requirements; (4) develop rigorous research designs and submit updated field study protocols and research instruments to the IRB; and (5) ensure multi‐institutional compliance with the IAA process. Our leadership team met monthly to review progress and to develop research protocols and shared documents for tracking researcher status.

### 4.2 Building Relationships

In early 2015, soon after funding was approved for the NIST‐CoE, we reached out to the Research Integrity and Compliance Review Office at Colorado State University to discuss best practices for ethics compliance considering the longitudinal scope of the project and the number of researchers involved. This was an important initial step because according to IRB requirements, funding should not be allocated, and research should not begin until projects involving human subjects have an established protocol in place. It is therefore essential to develop and build such relationships with compliance officers early in the research process (Packenham et al., 2017), while also working together to identify and understand the distinct ethical dynamics of research in disaster settings (Browne & Peek, 2014; Falb, Laird, Ratnayake, Rodrigues, & Annan, 2019).

Early meetings with the Colorado State University IRB helped us articulate the research goals and objectives for the field study component of the project. We also came to better understand the ethics and protocol requirements for the IRB review and the arrangement for the IAA. Because this research is federally funded and NIST researchers participate in many of the core tasks, NIST IRB officials also attended these early meetings. This helped to align the expectations of our academic institutions and the federal mission agency partner. These meetings also led us to develop a process for monitoring the completion of CITI training among the field research team and establish a timeline for submitting and approving the core protocol and amendments specific to each field study.

### 4.3 Educating the Team

Most of the researchers involved in the CoE are engineers who were not familiar with ethics training, human subject protocols, or IRB processes. Although the social scientists on the team all had previous IRB experience, it was critical to educate the entire team about the importance of conducting ethically informed research (O'Mathúna, 2009) and about the IAA approval process that we would be following with Colorado State University as the lead institution. We found that researchers across disciplines, including social scientists, had little or no experience with the IAA process during previous studies. Therefore, the leadership team regularly communicated with all researchers about ethics processes and IRB and IAA requirements during NIST‐CoE semi‐annual in‐person meetings, as well as through more informal channels such as telephone calls and emails with team leads at each institution.

### 4.4 Writing the Base Protocol and Gaining IRB Approval

The National Institutes of Health (NIH) has made significant strides in creating and promoting protocols for single institutional reviews for multisite research (NIH, 2016). These changes are notable for the hazards and disaster field because postdisaster rapid response studies often require that investigators enter the field soon after an event occurs to collect perishable data (Gaillard & Peek, 2019; Louis‐Charles et al., 2020; Oulahen et al., 2020). As previously noted, IRB's can take weeks or months to approve a submitted protocol (Liberale & Kovach, 2017). To address the need for ethics oversight and compliance, as well as contingent approval status for time‐sensitive disaster studies that involve multiple institutions (Miller et al., 2016; Packenham et al., 2017), our team developed a NIST‐CoE “core research protocol” that could be expanded on by submitting amendments for all future field studies. The lead authors of this protocol, which has been published, all had extensive experience with rapid response disaster research (Tobin et al., 2021). This core protocol was created using the Colorado State University IRB form and required supplemental documents, including recruitment scripts, interview guides, and a survey instrument. The protocol and associated research documents used placeholder text that could be amended to specify the exact location of the study, number of participants, specific research questions, and any updated forms or instruments based on the intent of the study and actual disaster context (Peacock et al., 2020).

After submitting the core research protocol for approval, we were required to undergo a full board review with the IRB committee at Colorado State University. This allowed the IRB to ask our team clarifying questions and for us to respond verbally and later via written revisions based on their recommendations. Our core research protocol was approved in the spring of 2016, with the written understanding that the NIST‐CoE would submit amendments in the event of an actual disaster event that we planned to study. Given the time sensitive nature of rapid response disaster research, the Colorado State University IRB committee agreed to review and approve or disapprove any disaster‐specific amendments within 48 hours of submission. The NIST‐CoE research team committed to respond to requests for clarification or revision in a similarly timely manner. The approved IRB protocol states that access to primary data collected in the field is limited to investigators who have completed the CITI training and whose universities have signed the IAA agreement.

### 4.5 Testing and Updating the Protocol

In July 2016, the NIST‐CoE had the opportunity to test the approved core protocol by conducting a hindcast (which is an approach to test mathematical simulations by comparing known results with predicted outputs from a computational model) of Joplin, Missouri, following the tornado that had occurred there in 2011 (see Attary et al., 2018; Attary, van de Lindt, Mahmoud, & Smith, 2018). Because five years had passed since the disaster—rather than days or weeks, which is more typical in rapid response disaster field studies—we had more time to evaluate what was needed to update the protocol. Following best practice guidance (Nuffield Council on Bioethics, 2020), we ensured that those updating the protocol had previous research experience in Joplin and extensive local contacts in the study site. On July 8, 2016, our field study leadership team submitted an amendment to the core protocol, which was assessed within 24 hours by the Colorado State University IRB review team. We addressed all required revisions, and the amendment was approved on July 12, 2016—this was only a four‐day window from submission to approval. The research team entered the field and began collecting data on July 19, 2016.

After Hurricane Matthew made landfall in South Carolina on October 8, 2016, the protocol was comprehensively put into action for an interdisciplinary investigation that became a multiyear longitudinal study to systematically document the recovery of Lumberton, North Carolina (van de Lindt et al., 2018, 2020). On November 16, 2016, the field study team submitted an amendment to the Colorado State University IRB. The IRB responded within two days and then the NIST‐CoE team began addressing the required revisions. The amendment was approved as revised on November 23, 2016, and the team entered the field on November 27, 2016. The turnaround times highlighted here would not have been possible without having had the core protocol in place.

Our team also took additional steps to help ensure the robustness of the revised protocol. For example, before submitting the IRB amendment to study Hurricane Matthew, our research team gathered as much information about the disaster event as possible through a search and analysis of news articles, government reports, and other nonprimary data sources (Tobin, 2020). This allowed the team to build a protocol amendment that highlighted the impacts of the event, the sociocultural context of the community where the disaster occurred, and the rationale for the research questions, sampling procedures, and proposed data collection plan. In amending a field study protocol to a specific context, it is also important to identify a group of potential researchers from the broader project team. In our case, researchers were selected for the field study effort based on eight *a priori* criteria that included: (1) completion of the required CITI ethics training; (2) completion of the field research workshops hosted by our field studies leadership team; (3) proximity to and familiarity with the disaster site; (4) availability enter the field simultaneously with other team members; (5) area of expertise relevant to the disaster context; (6) interest in the disaster event; (7) cultural competence and knowledge of the affected geographic region; and (8) the principal investigators’ judgment on funding availability and appropriate team composition.

The NIST‐CoE field study team continues to track community recovery and resilience through repeated research trips to Lumberton, North Carolina, with the most recent visit occurring in April 2019. As this project progresses, the Colorado State University IRB has required that steps are taken to ensure consistency and updates to ethics training and protocol review. The CITI ethics training that was required for all researchers expired after three years and therefore needed to be renewed for members to continue conducting field studies or working with any of the data produced by them. In addition, the IRB requires the research team to submit a review annually that updates the IRB on information such as the number of participants that have been involved in the study, the number of field studies left to be completed, and updated plans for data analysis, management, and publication. Each year after the review form is approved by the Colorado State University IRB, all associated documents and approval forms needed to be submitted to the institutions covered under the IAA.

## 5 CONCLUSION

This summary of the IRB process serves as a potential model for large, multiinstitution, interdisciplinary research projects. Following current guidance, we highly recommend that more universities that embark on cooperative interdisciplinary disaster research projects use joint review agreements through IAAs to increase ethical standards, reduce the burden to participants, and streamline efforts to get well‐trained researchers into the field rapidly when a disaster occurs (Miller et al., 2016; Packenham et al., 2017). In light of the 2018 updates to the human subjects protection regulation that mandates single‐IRB review for collaborative research, this article serves as a case study for how the single‐IRB review process can be accomplished in an efficient manner for time‐sensitive postdisaster field research (U.S. Department of Health and Human Services, 2018).

The trend of IRBs moving into a space where they approve disaster field studies contingent on postdisaster amendments is a significant advancement for the field of disaster studies and research ethics in general. We encourage all disaster research teams to begin building relationships with their IRBs in the early stages of project conceptualization and development to ensure that the goals of the project can be met with minimal delays from human subjects approval boards and maximum focus on reducing study participant risk. We also recommend the establishment of an interdisciplinary leadership team that is specifically focused on ensuring that all IRB requirements are understood and met by all researchers who are part of the larger team. This leadership team can also take the lead in managing IRB and IAA updates and ensuring the ethical collection and use of field study data. This, in turn, can advance evidence informed disaster risk reduction and resilience efforts in communities.

## ACKNOWLEGMENTS

This research was conducted as part of the National Institute of Standards and Technology (NIST) Center of Excellence for Risk‐Based Community Resilience Planning under Cooperative Agreement 70NANB15H044 between NIST and Colorado State University. The content expressed in this article are the views of the authors and do not necessarily represent the opinions or views of NIST or the U.S. Department of Commerce. The author team gratefully acknowledges Evelyn Swiss at the Colorado State University Institutional Review Board; Sara Hamideh and Elaina Sutley for their collaboration; Jessica Austin, Kamryn Roper‐Fetter, and Zoe Welz for their research assistance; the anonymous reviewers for their feedback; and Karen Lowrie for her patience and support throughout the publication process.
